# How to reduce the costs of ornaments without reducing their effectiveness? An example of a mechanism from carotenoid-based plumage

**DOI:** 10.1007/s00265-016-2090-6

**Published:** 2016-02-29

**Authors:** Adrian Surmacki, Anastazja Ragan, Ziemowit Kosiński, Marcin Tobółka, Paweł Podkowa

**Affiliations:** Department of Avian Biology and Ecology, Faculty of Biology, Adam Mickiewicz University, Umultowska 89, 61-614 Poznań, Poland; Institute of Zoology, Poznań University of Life Sciences, Wojska Polskiego 71C, 60-625 Poznań, Poland

**Keywords:** *Bombycilla garrulus*, Courtship behavior, Color expression, Individual condition, Male display, Sexual secondary traits

## Abstract

**Abstract:**

Carotenoid-based ornaments are often considered to be honest indicators of individual quality assessed by potential mates. However, males can use a variety of strategies that minimize the amount of costly carotenoids used while retaining the effectiveness of color signaling. Birds could do this by altering pigment intake, metabolism, or its presentation to a potential signal receiver. Here, we propose a new mechanism of lowering the costs of carotenoid displays in birds: differential allocation of pigments within single feathers. We studied the coloration of the yellow terminal tail bands of rectrices of male Bohemian waxwings. Using reflectance spectrometry, we show that the two central rectrices are most intensively colored compared to other rectrices. More detailed analyses reveal that these differences result from feather-specific patterns of rectrices coloration. The outer feather vanes of the outermost rectrices are more intensively colored compared to the inner vanes. However, the central rectrices have equally colored vanes that are, on average, more intensively pigmented than the outermost rectrices. When the waxwing tail is folded, the outermost rectrices are covered by other feathers, except for the narrow, outer vane. Central rectrices, however, form the outermost layers which are not obscured by other tail feathers. Thus, the feather vanes that are the most visible to potential viewers are also the most pigmented. These results support the occurrence of a previously overlooked mechanism to reduce the costs of carotenoid-based ornaments: precise pigment distribution to maximize efficiency of signals within single feathers.

**Significance statement:**

Males of many bird species use bright carotenoid-based plumage coloration to attract females. These traits are physiologically expensive such that only individuals in prime condition can develop the most vivid colors. Males often “cheat” to obtain attractive appearances at lower costs. We showed that this goal could be achieved by differential deposition of pigments into the most conspicuous feather regions. Bohemian waxwing males have yellow tips on their rectrices of which the outer vanes are more brightly colored compared to the inner vanes. These inner feather vanes are usually covered by other feathers and are, thus, less visible to conspecifics. The only exception is the pair of central rectrices that are fully exposed, and both feather vanes are equally colored. In this species, males minimize the use of costly carotenoid pigments while maintaining elaborate ornamentation of plumage regions that are most visible to potential mates.

## Introduction

In many animal species, the evolution of showy secondary sexual characters in males is thought to be driven mainly by female choice (Andersson 1994; but see examples of intraspecific signal evolution of ornaments, e.g., Ninnes and Andersson 2014; Ninnes et al. 2015). These characters include integument coloration (e.g., Kodric-Brown 1985; Hill 1991), behavioral displays (e.g., Barske et al. 2011), courtship vocalization (e.g., Tomaszycki and Adkins-Regan 2005), morphological structures (Andersson 1982), and many others. The central assumption of honest advertisement models of sexual selection is that ornaments are costly to produce (Zahavi 1975, 1977). Numerous studies have shown that when an animal’s condition is reduced by environmental factors (e.g., nutritional and oxidative stress, pathogens, or increased parental effort), the quality of ornamentation subsequently decreases (Griffith 2000; Nowicki et al. 2004; Hill 2006). However, tactics used by males to reduce ornament production costs may weaken the linkage between ornament quality and condition (Hill 1994; Badyaev 2004). This process should induce sexual conflict because females should seek cues that accurately reflect male quality (Hill 1994).

Carotenoid plumage is an example of costly ornamentation, and males could benefit by expressing it at reduced costs. Carotenoids are acquired solely through diet, and animals experience trade-offs between allocating pigments into the integument coloration versus toward other important physiological functions, like cellular respiration (Hill 2006, 2014). Hill (1994) and Badyaev (2004) reviewed several strategies used by males to reduce the cost of carotenoid-based ornaments. These strategies can be divided into two classes. The first class is associated with carotenoid acquisition and metabolism. For example, males may adjust their diet to maximize carotenoid intake during the time of ornamental plumage growth (McPherson 1988) that could be accomplished by selectively choosing carotenoid-rich food or a food containing carotenoids that can be directly deposited into ornaments (Badyaev 2004). Alternatively, birds can selectively metabolize only the carotenoids that are used for pigmentation, a strategy observed in flamingos (*Phoenicopterus* spp.), orioles (*Icterus* spp.), and scarlet ibises (*Endocimus ruber*) (Hill 1994; Badyaev 2004 and papers cited therein). In the second strategy class, males alter the expression of carotenoid ornaments by maximizing the display of pigments (Hill 1993). For example, confining a pigmented plumage area to a small patch can lead to an increase in color intensity or feather structure can be modified such that the coloration is displayed in the most efficient manner (Hill 1994). Although examples of such mechanisms were described decades ago (Hill 1994 and papers cited therein), this process has yet to be quantitatively described.

We propose a new mechanism for lowering the costs of carotenoid-based plumage displays in birds: males produce more elaborate coloration on the feathers and feather parts that should be the most visible to the signal receivers. We suggest that reduced production costs could be obtained by uneven incorporation of carotenoids over ornaments. Less conspicuous ornament areas may receive less pigment that, instead, may be used in important physiological processes like immune defense and/or could be diverted to ornamentation of the most visible ornament areas.

Our model species is the Bohemian waxwing (*Bombycilla garrulus*), a species with multiple carotenoid-based ornaments: a yellow terminal tail band; yellow, oval spots on the tips of the primary feathers; and red, waxy appendages on the secondary feathers (Svensson 1992). Although these carotenoid ornaments occur in both sexes, males display larger appendages and yellow patches that are more intensively colored (Svensson 1992). A clear dichromatism in carotenoid ornaments suggests that these traits could evolve as a result of female mate choice in Bohemian waxwings. Female preferences for red appendages have been demonstrated in the closely related species, cedar waxwing (*Bombycilla cedrorum*, Mountjoy and Robertson 1988). Here, we focused on the yellow tail band of males that is pigmented with canary xanthophylls A and B (McGraw 2006).

## Methods

To minimize observer bias, blinded methods were used during material collection and subsequent measurements and analyses. All birds were collected dead after collisions with buildings in Poznań, western Poland, in winters of 2004 and 2008. Sex was determined by morphological features (Svensson 1992), and visual assessment of reproductive organs during dissection and male age was determined by feather morphology (Svensson 1992). In this study, we used 35 yearling males. Thus, the plumage ornaments that we studied were grown while the male was a juvenile (nestling or fledgling). However, yearling Bohemian waxwings may breed and perform courtship displays similar to adults (Cramp 1998); it is therefore reasonable to assume that their colorful feathers may play a role in sexual selection. Before dissection, all 12 rectrices were plucked and stored in plastic bags in the dark until further measurements.

### Reflectance measurements

We measured the reflectance of the distal tips of the yellow rectrices using a USB4000 spectrometer connected to a pulsed xenon lamp PX2 (Ocean Optics, Dunedin, FL, USA) with a bi-furcated fiber optic measuring probe FCR-7UV200-2-1.5x100 (Avantes, Apeldoorn, The Netherlands). The probe was held at a 90° angle to the feather surface and illuminated an area of ca. 1 mm in diameter. Before measuring the feathers, we standardized measurements using a white standard (WS-1-SL, Labsphere, North Sutton, NH, USA) while the dark standard was taken by turning off the light source and covering the probe. Spectral measurements were expressed as percent reflectance of light per wavelength in relation to a white standard reflectance (100 %). We measured reflectance of all 12 rectrices. We took six readings from the dorsal side of yellow tip in each rectrix, including three readings per inner and outer vane.

We processed all the spectral data using RCLR v0.9.28 software (Montgomerie 2008). For each individual, we calculated the carotenoid chroma ((*R*_450_ − *R*_700_) / *R*_700_; Montgomerie 2008), which is a good predictor of carotenoid content in yellow feathers (Peters et al. 2004). We transformed chroma values into positive values that are intuitively more appropriate; the higher values express a higher carotenoid chroma. In the analyses of between-feather color variation, we used a carotenoid chroma averaged for the entire dorsal side of the vane. In remaining analyses, we used a carotenoid chroma averaged separately for the inner and outer vane.

### Statistical analysis

We used ANOVA for repeated measurements to test for differences in the average carotenoid chroma between yellow tips of all rectrices. In this case, 12 repeated measurements were compared. We applied the same method to test for differences in the coloration between the outer and inner feather vanes in right outermost (R1) and right central (R6) rectrices. In total, four repeated measurements were compared. In both analyses, we used the Tukey honest significant difference (HSD) test for post hoc comparisons.

## Results

**Fig. 1:**
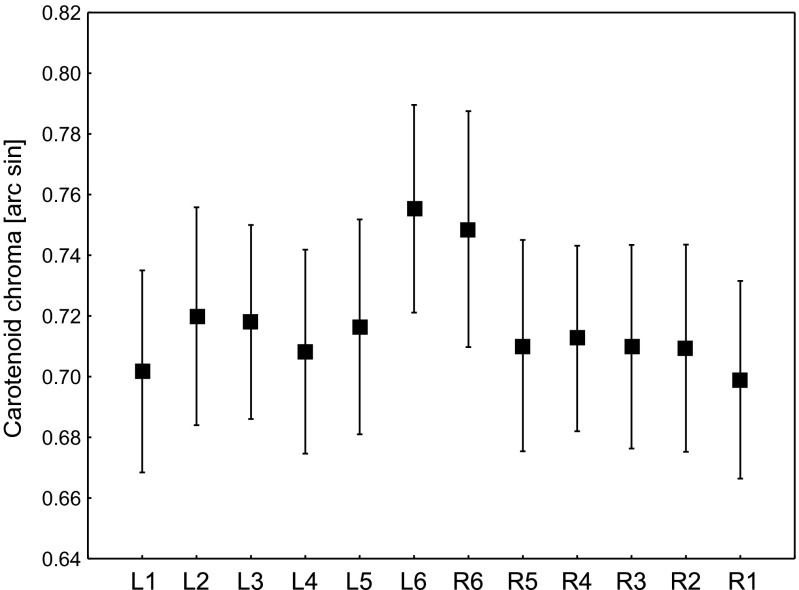
Carotenoid chroma of the yellow tips of the rectrices of yearling male Bohemian waxwings (mean ± 95 % CF). *Symbols on the x-axis* refer to rectrices on the left (*L*) and right (*R*) sides of the tail. Rectrices 1 and 6 are the outermost and innermost tail feathers, respectively

There were significant differences in the carotenoid chroma between rectrices within the same individual (*F*_11, 374_ = 7.62, *p* < 0.001; Fig. 1). Post hoc tests revealed that both central rectrices (i.e., L6 and R6) exhibited a significantly greater carotenoid chroma compared to all others (Table 1); however, the differences between L2 and R6 were only marginally significant (Table 1). There were no statistically significant differences in the chroma between L6 and R6 rectrices and between all remaining rectrices (L1–L5, R1–R5; Table 1).

**Table 1:** Results of Tukey HSD tests comparing values of carotenoid chroma of the yellow tips of the rectrices of yearling male Bohemian waxwings

Rectrix	L2	L3	L4	L5	L6	R6	R5	R4	R3	R2	R1
L1	0.657	0. 796	1.000	0.888	**	**	0.998	0.988	0.999	0.999	1.000
L2		1.000	0.977	1.000	*	0.054	1.000	1.000	0.993	0.990	0.431
L3			0.994	1.000	*	*	0.999	1.000	0.999	0.998	0.586
L4				0.999	**	**	1.000	1.000	1.000	1.000	0.997
L5					**	*	1.000	1.000	1.000	1.000	0.715
L6						1.000	**	**	**	**	**
R6							**	*	**	**	**
R5								1.000	1.000	1.000	0.983
R4									1.000	1.000	0.930
R3										1.000	0.987
R2											0.991

For explanations of symbols, see Fig. 1. Values in each cell are *p* values for a given pair of comparisons

**p < 0.001; *p < 0.05

**Fig. 2:**
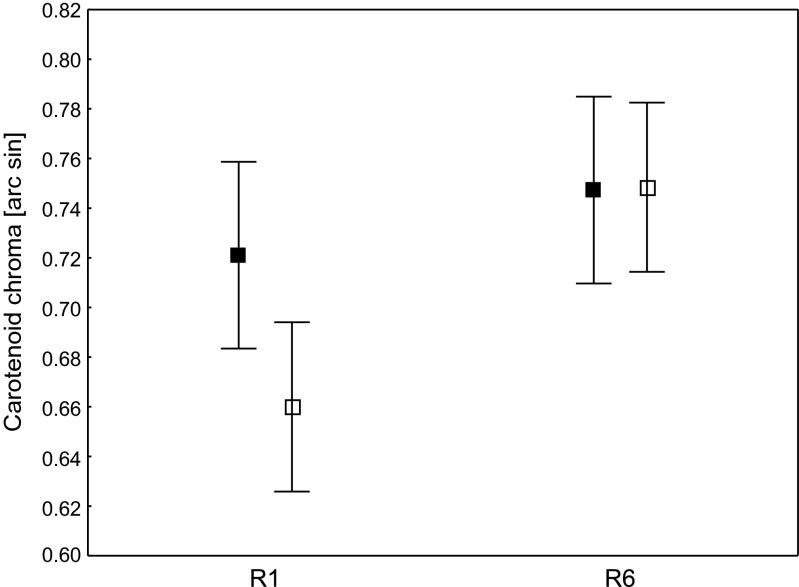
Carotenoid chroma of the outer and inner vanes of the yellow R1 and R6 rectrices of yearling male Bohemian waxwings. *Filled square* outer vane; *empty square* inner vane

**Fig. 3:**
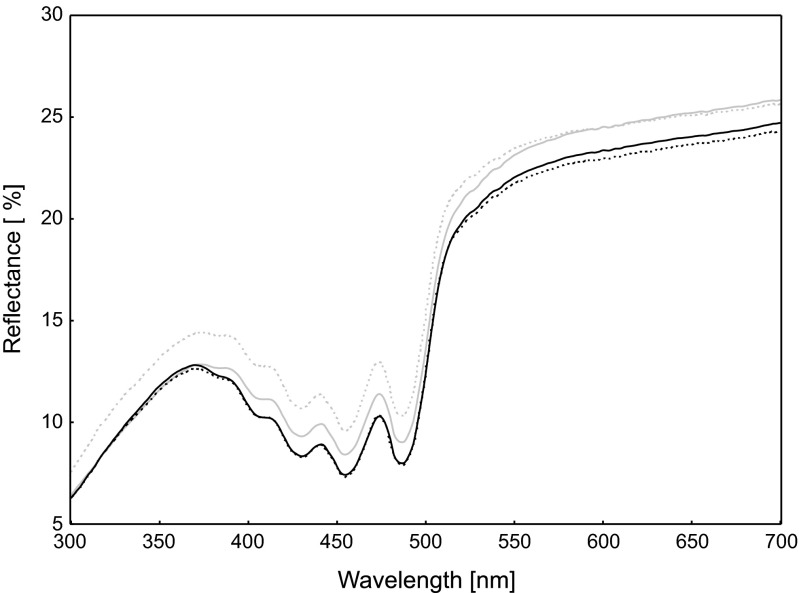
Mean reflectance spectra of the yellow tips of the rectrices of male Bohemian waxwing. The *gray and black lines* refer to R1 and R6 rectrix, respectively. *Solid and dashed lines* refer to outer and inner feather vanes, respectively

Carotenoid chroma differed significantly between feather vanes (outer and inner) and amongst rectrices (R1 and R6; *F*_3, 102_ = 23.25, *p* < 0.001; Figs. 2 and 3). We found a significantly greater carotenoid chroma in the outer vanes compared to the inner vanes in the R1 rectrix (Tukey HSD test; *p* < 0.001; Figs. 2 and 3). In contrast, inner vanes and outer vanes were equally chromatic in the R6 rectrix (Tukey HSD test; *p* = 1.00; Figs. 2 and 3). The carotenoid chroma of the outer and inner vanes of the R6 rectrix was significantly higher when compared to the inner vanes of the R1 rectrix (Tukey HSD test; *p* < 0.001 in both cases; Figs. 2 and 3), but no differences were found when compared to the outer vanes of the R1 rectrix (Tukey HSD test; *p* = 0.14 and *p* = 0.12, respectively; Figs. 2 and 3).

## Discussion

Our results demonstrate that, within the same pigment ornament, coloration is significantly differentiated between feather types and feather vanes. Several lines of evidences suggest that this differentiation could evolve as a consequence of an evolutionary strategy to lower the costs of male ornament production while simultaneously maintaining its efficiency as a visual signal. Our study provides the first evidence of this mechanism.

**Fig. 4:**
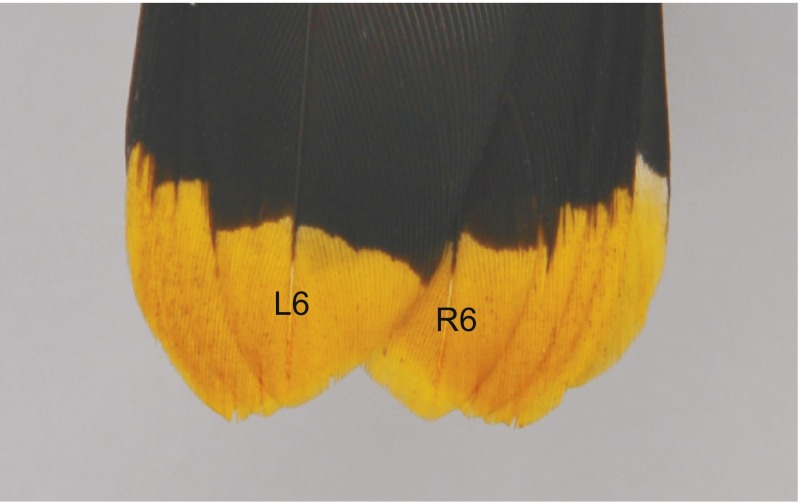
A photograph showing the positions of the rectrices on the dorsal side of the tails of Bohemian waxwings (the two central feathers (*L6* and *R6*) are labeled)

Both within- and between-feather differences in the carotenoid chroma in waxwing males could be explained by their relative conspicuousness. When the tail is folded, the two central rectrices (L6 and R6) form the outermost and uncovered layer (Fig. 4). Except for the narrow strip of the outer feather vane, the remaining rectrices (L1–L5 and R1–R5) are covered by feathers (Fig. 4). We demonstrate that waxwing males produce more elaborate color (richer carotenoid chroma) on the outer feather vane that is more often visible. The only exceptions are the two central rectrices (R6 and L6), but these are always exposed to potential signal receivers. In this case, both feather vanes are equally colored and are on average more saturated compared to the other rectrices. Some features of Bohemian waxwing courtship behavior suggest that females may assess male quality based on the characteristics of the dorsal surface of the folded tail (Cramp 1998). Paired birds perch very close to each other, and the male assumes a generally horizontal position with the tail depressed (Cramp 1998). Also, displaying males bend their closed tail laterally toward the female (Cramp 1998).

Because our study is correlative, we cannot rule out other, non-adaptive, explanations for the color pattern of waxwing rectrices. Research on several other species, including cedar waxwings, indicates that yellow, carotenoid-based coloration is a product of both pigment deposition and feather microstructure (Shawkey and Hill 2005). It is therefore possible that differences in Bohemian waxwing tail coloration could be, at least partially, a by-product of keratin organization in barbs. However, recent studies have revealed that carotenoid-based coloration is more influenced by a variation in carotenoid content than feather microstructure (Shawkey et al. 2006; Jacot et al. 2010). Thus, a variation in feather structure probably does not explain differences in the coloration between rectrices and their vanes in Bohemian waxwing males. Another possible explanation of between-feather variation in carotenoid chroma could be a differential rectrix growth rate or (and) an order of rectrix growth. However, in nestling passerines, rectrices grow simultaneously (Shirihai et al. 2010), so their growth rate and growth sequence are unlikely to explain differences in carotenoid coloration. Moreover, the yellow spots cover only ca. 5 mm of the distal tip of the rectrix (ca. 7 % of the total feather length). If growth rate varies between feathers, it seems unlikely that the region of the feather that erupts from feathers first would be affected.

Numerous studies have demonstrated that carotenoid-based traits bear important condition-dependent costs for their owners (reviewed in Hill 2006). Environmental agents like access to dietary pigments, parasite infections, and nutritional state are the most important factors influencing plumage coloration change in the subsequent molt (reviewed in Hill 2006). Moreover, carotenoid-based nuptial plumage coloration can be influenced by carotenoid or energy trade-offs associated with the use of carotenoids to either produce ornaments or aid in physiological processes (e.g., molt, Serra et al. 2007) or behavioral activities (e.g., parental care, Hill 2002). According to the most recent hypotheses, vital cellular processes like mitochondrial respiration create a strong linkage between expression of carotenoid-based colors and various aspects of animal condition (Hill and Johnson 2012; Johnson and Hill 2013; Hill 2014). Not surprisingly, males tend to reduce the costs of carotenoid color development without sacrificing their attractiveness. Mechanisms based on “pigment presentation” in a feather are especially important because they directly affect how females perceive the color signal. One of tactics used by males is to concentrate pigments into a small, but intensively colored, plumage area. The example is the extent of ventral carotenoid-based coloration in the *griscomi* subspecies of the house finch (*Haemorhouse mexicanus*), which show more concentrated carotenoid pigments in a smaller patch area compared other populations of house finches (Hill 1993; Badyaev 2004). Other mechanisms to maximize carotenoid ornament display are based on modifications of feather structure. Barbs containing pigments may be laterally compressed, so their broad sides are oriented perpendicularly to the surface of the feather (Brush and Seifried 1968; Olson 1970; Hudon 1991). The function of this modification is to increase chroma and to shift hue toward longer wavelengths (Brush and Seifried 1968; Olson 1970; Hudon 1991). Furthermore, carotenoid-containing barbs have reduced barbules to enhance transmission of colored light (Brush and Seifried 1968; Olson 1970). This kind of carotenoid-pigmented feather modification has been described in several species, for example Gouldian finches (*Poephila gouldiae*, Brush and Seifried 1968), Guianan cock-of-the-rock (*Rupicola rupicola*, Olson 1970), and western tanager (*Piranga ludoviciana*, Hudon 1991).

Compared to the above examples, the proposed mechanism of carotenoid display maximization revealed in waxwing males seems to be highly fine-tuned. It is based on a very precise distribution of pigment within a small area of a single feather. Moreover, the pattern of within-feather coloration depends on the location of the feathers on the bird’s body. Birds probably do not simply confine yellow coloration to the outer feather vane of the one to five rectrices because inner feather vanes are also sometimes visible, depending on the extent to which the tail is spread.

Although yearling Bohemian waxwings breed and use carotenoid-based ornaments during courtships (Cramp 1998), similar studies focusing on females and older males are warranted. Considering that production of structural and melanin-based colors may also be physiologically costly (e.g., Peters et al. 2007; Siefferman and Hill 2007; Galván and Solano 2015) and that, in many avian species, colorful ornaments are located on rectrices (e.g., greenfinch *Chloris chloris*, Hõrak et al. 2004), the mechanism reported here might be quite common and not restricted to carotenoid-based coloration. The strategy of feather vane-specific pigment investment could be easily tested in carotenoid supplementation experiments. We would expect that individuals facing deficiency of pigments in their diet should locate them more specifically in most visible feathers or feather parts. In contrast, when carotenoids are abundant, the within-ornament variance may be expected to be less extreme.
